# Recent advancements in traditional Chinese medicine for COVID-19 with comorbidities across various systems: a scoping review

**DOI:** 10.1186/s40249-024-01263-8

**Published:** 2024-12-19

**Authors:** Xiyu Shang, Yuqing Cao, Yang Guo, Lei Zhang, Jiajia Li, Huifang Zhang, Yipin Fan, Yuxuan Huang, Jiantao Li, Yanping Wang, Yibai Xiong, Qiujie Cai, Huamin Zhang, Yan Ma

**Affiliations:** 1 Institute of Basic Research in Clinical Medicine, China Academy of Chinese Medical Sciences, Beijing, 100700 China; 2 Institute of Traditional Chinese Medicine Information, Chinese Academy of Traditional Chinese Medicine, Beijing, 100700 People’s Republic of China; 3 Fuwai Hospital, Chinese Academy of Medical Sciences, Beijing, 100037 China; 4 NHC Key Laboratory of Human Disease Comparative Medicine, Beijing Key Laboratory for Animal Models of Emerging and Remerging Infectious Diseases, Institute of Laboratory Animal Science, Chinese Academy of Medical Sciences and Comparative Medicine Center, Peking Union Medical College, Beijing, 100021 China; 5 Institute of Basic Theory for Chinese Medicine, China Academy of Chinese Medical Sciences, Beijing, 100700 China

**Keywords:** Traditional Chinese medicine, COVID-19, Comorbidities, Clinical effect, Review

## Abstract

**Background:**

Traditional Chinese medicine (TCM) has developed a rich theoretical system and practical experience in fighting to infectious diseases over the past thousands of years, and has played an important role in controlling the spread owing to its unique advantages. In particular, its significant contribution to the prevention and control of Corona Virus Disease 2019 (COVID-19) is widely recognized. COVID-19 infection is mainly non-severe with a favorable overall outcome, but patients with comorbidities tend to have a poor prognosis. However, a comprehensive review of TCM for preventing and treating COVID-19 with comorbidities across various systems is still lacking. Hence, this scoping review aims to conduct a comprehensive investigation on treatment outcome of TCM for treating COVID-19 with comorbidities across various systems.

**Methods:**

The scoping review was conducted by searching English databases including PubMed and Web of Science, and Chinese databases including China National Knowledge Infrastructure and Wanfang between January 2020 and January 2024. We followed the inclusion and exclusion criteria to identify relevant literature. Information for inclusion in the literature were subsequently extracted and consolidated.

**Results:**

We enrolled 13 literature that met the inclusion criteria in the review finally. Our analysis revealed that research on COVID-19 with comorbidities was mostly focused on circulatory diseases, including hypertension, heart failure, and cerebrovascular diseases, most common comorbidities were hypertension. Followed by endocrine and metabolic diseases such as diabetes, respiratory diseases including pulmonary tuberculosis and chronic obstructive pulmonary disease have been also addressed. However, there were few studies on co-infectious urogenital system disease, and no studies on the rheumatic, immune, hematological, nervous, reproductive, and skin systems diseases. Based on existing studies, TCM has significantly improved the clinical symptoms of COVID-19 with comorbidities such as fever, fatigue, dry cough, anorexia and asthma, the absorption of lung lesions, shortened the duration of viral shedding and the course of disease.

**Conclusions:**

TCM has great application prospects in treating COVID-19 with comorbidities. These findings could provide important evidence for clinicians to treat COVID-19 with comorbidities. Multi-center studies are required to confirm our results in the future.

**Graphical Abstract:**

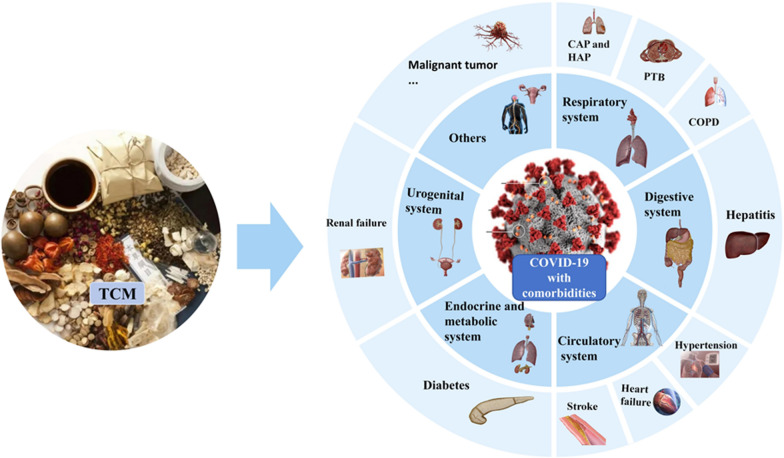

**Supplementary Information:**

The online version contains supplementary material available at 10.1186/s40249-024-01263-8.

## Background

Traditional Chinese Medicine (TCM) has thousands of years in fighting infectious diseases and has played a vital role in epidemic control. Its significant contributions to the management of Coronavirus Disease 2019 (COVID-19) have been widely recognized [1–6]. The World Health Organization declared that COVID-19 is no longer a public health emergency of international concern on May 5, 2023. However, the threat of COVID-19 to humans persists with the severe acute respiratory syndrome coronavirus 2 (SARS-CoV-2) continues to mutate. For instance, there have been over 770 million cases and 7 million deaths globally. Notably, in the past 28 days (July 1 to July 28, 2024), there were over 200,000 new cases, 27,000 new hospitalizations, and over 3300 deaths [7]. While COVID-19 primarily presents as a non-severe illness with favorable prognosis, patients with comorbidities tend to have poorer outcomes. Studies indicated that the mortality rate among these patients can be as high as 65.9% [8]. Comorbidity refers to the clinical state in which one or more diseases coexist alongside the primary disease. Approximately 29.4% of COVID-19 patients have at least one comorbidity, and those with comorbidities experience more severe symptoms, increased rates of severe illness, and a higher risk of death compared with those without comorbidities [9–15].

Previous studies reported that TCM interventions, such as Qingfei Paidu decoction (QFPDD) and Maxing Shigan decoction were demonstrated good efficacy and safety in treating COVID-19 [16–19]. Additionally, TCM showed favorable treatment outcomes for COVID-19 patients with comorbidities such as chronic hepatitis B, cardiovascular and cerebrovascular diseases [17, 20]. However, no comprehensive review has been conducted on the role of TCM in the prevention and treatment of COVID-19 among patients with comorbidities across different systems. This knowledge gaps might limit the clinical application of TCM in managing COVID-19 with comorbidities. Therefore, it is urgent to conduct a comprehensive and systematic review of TCM’s efficacy in treating COVID-19 with comorbidities across various systems, thereby providing higher-level evidence to promote its clinical applications. In this study, we reviewed relevant literature and summarized the efficacy of TCM in treating COVID-19 patients with comorbidities affecting systems such as the circulatory, endocrine, metabolic, respiratory, and digestive systems. The findings aim to enhance the understanding of TCM's current situation in managing COVID-19 with comorbidities and provide further reference for clinicians to ensure rational applications.

## Methods

### Search strategy

In the present study, two reviewers independently searched the literature on COVID-19 with comorbidities across various systems using four databases, including two English-language databases such as PubMed (https://www.ncbi.nlm.nih.gov/pubmed/) and Web of Science (https://www.webofscience.com/), and two Chinese databases such as China National Knowledge Infrastructure (CNKI, https://www.cnki.net/) and Wanfang Data (https://www.wanfangdata.com.cn/), covering the period from January 1, 2020 to January 31, 2024. The structured search strategy combined the following MeSH terms and keywords: Huashi Baidu, HSBD, Xuanfei Baidu, XFBD, Jinhua Qinggan, JHQG, Lianhua Qingwen, LHQW, Xuebijing, XBJ, Qingfei Paidu decoction, Lung Cleansing & detoxifying decoction, Lung Cleansing detoxifying decoction, QFPDD, traditional Chinese medicine, TCM, oriental medicine, Chinese Medicine; COVID-19, covid 19, sars cov 2, SARS-CoV-2, coronavirus 2019, coronavirus disease 2019, Novel coronavirus, 2019-ncov; treatment, effective, effect, outcome, follow up, efficacy, therapeutic effect. The search terms used are listed in Additional file 1 (Table S1A–B).

### Inclusion criteria and exclusion criteria

EndNote X9 (Clarivate, Philadelphia, USA) was used to remove duplicate articles. Then, the titles and abstracts of articles were read to filter out those not relevant to the research objectives. Full texts were carefully evaluated according to the following inclusion criteria: (1) the studies on TCM for COVID-19 with comorbidities; (2) the studies are clinical studies including randomized controlled trials (RCTs), non-RCTs, and case reports. The exclusion criteria were as following: (1) unobtainable in full text format; (2) the literature described complications of COVID-19; (3) the literature did not present treatment outcomes; (4) in cases of duplicate data or publications, only the one with the most complete data or the earliest publication was incorporated.

### Classification of various disease systems

We defined the systems based on the reference of *Systematic Anatomy* (9th edition) [21] and categorized different diseases according to the International Classification of Diseases, Eleventh Revision (ICD-11) (https://icd11.pumch.cn/). In this review, we demonstrated various disease systems including circulatory system, endocrine and metabolic system, digestive system, respiratory system, and urogenital system.

#### Circulatory system

The circulatory system includes the cardiovascular system and the lymphatic system. The cardiovascular system consists of the heart, arteries, capillaries, and veins, while the lymphatic system is composed of lymphatic vessels, lymphatic tissues, and lymphatic organs. Diseases of the circulatory system generally include hypertension, coronary heart disease, and heart failure (HF).

#### Endocrine and metabolic system

The endocrine system comprises endocrine glands and tissues. Endocrine, nutritional, or metabolic diseases generally include diabetes, malnutrition, overweight or obesity, hypoparathyroidism, and hyperparathyroidism.

#### Digestive system

The digestive system consists of the alimentary canal and the digestive glands. Diseases of the digestive system commonly include viral hepatitis, autoimmune hepatitis, and non-alcoholic fatty liver disease.

#### Respiratory system

The respiratory system consists of the respiratory tract and lungs. Diseases of the respiratory system generally include bronchitis, chronic obstructive pulmonary disease (COPD), asthma, and pneumonia.

#### Urogenital system

The urinary system includes the kidneys, ureters, bladder, and urethra, while the reproductive system comprises the internal and external genitalia. The internal genitals consist of the gonads, reproductive ducts, and accessory glands, while the external genitals include the organs of coitus between the sexes. Diseases of the genitourinary system generally include renal failure and glomerular disease.

### Quality assessment of included literature

The methodological quality of trials was assessed by two reviewers independently. RCTs were evaluated according to the Jadad scale, which consists of four items: randomization (0–2 points), double-blindness (0–2 points), and withdrawals (0–1 point). The scores were aggregated, with a total score of 0–1 indicating low quality, 2 indicating moderate quality, and 3–5 indicating high quality [22]. Non-RCTs were evaluated using the Methodological Index for Non-Randomized Studies (MINORS), which consists of 12 items, with a total score of 24. Scores were aggregated, where a total score below 8 points indicates low quality, 8–15 indicates moderate quality, and 16–24 indicates high quality [23]. The quality of case reports selected was assessed using the JBI Critical Appraisal Checklists, which consists of eight items [24].

### Data extraction and analysis

All retrieved literature was processed using EndNote X9 (Clarivate, Philadelphia, USA). Two reviewers independently extracted data, which included comorbidities, interventions, study type (single-center or multi-center), adverse reactions, outcomes, composition of TCM, and the usage of each TCM. Any discrepancies in the data extraction were reviewed and verified, with discussions facilitated by a third reviewer.

## Results

### General characteristics of studies

**Fig. 1:**
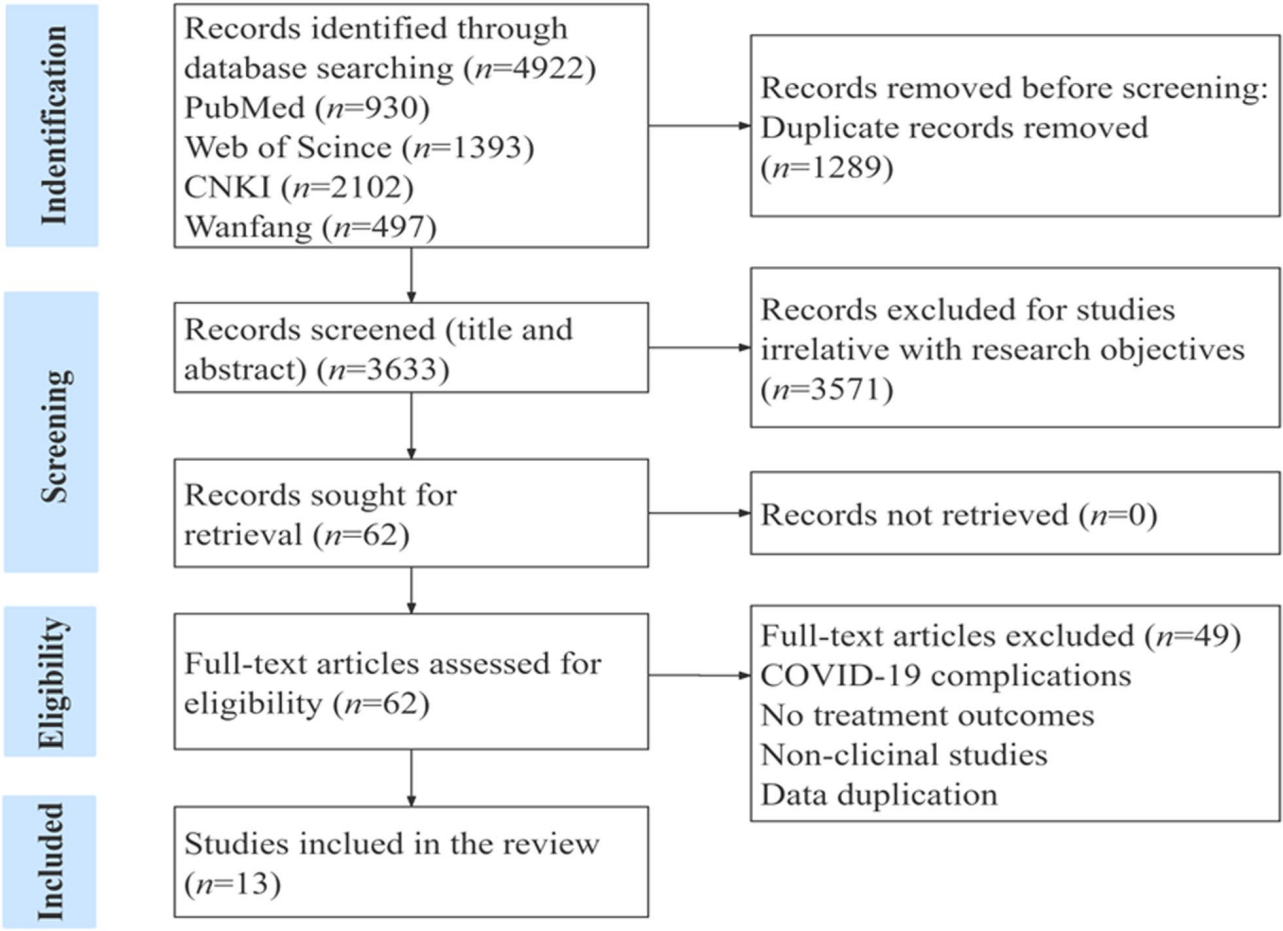
Flowchart of the study

We retrieved 4922 records totally, including 2599 and 2323 literature from Chinese and English databases, respectively. After removing duplicates, the titles, abstracts and full text of 3633 records were screened. Finally, we enrolled 13 studies including 3 RCTs, 8 non-RCTs, and 2 case reports. Flowchart of the study was shown in Fig. 1. Detailed search strategies were shown in Additional file 1 (Table S1 A–B).

Characteristics of the studies enrolled were seen in Table 1. To clearly present the TCM treatment regimens for fighting COVID-19 with various comorbidities, we also compiled the composition details of each TCM formula, including their Chinese, English, Latin names, as well as the respective doses of these herbs (Additional file 2: Tab S2).

**Table 1:** Characteristics of the studies enrolled

References	Comorbidities	Interventions	Single/ Multi-center	Study type	ADR	Limitations	Treatment outcomes
Li HZ et al. (2021) [16]	Hypertension	QFPDD and WMT	Multi	Non-RCT	No	Retrospective study	99.2% of patients were cured and discharged; one patient died
Li YZ et al. (2020) [30]	Hypertension	Qingfei decoction and WMT	Single	Non-RCT	No	Single-center, no parallel control group	No deaths
Zhang L et al. (2021) [20]	Cardiovascular and cerebrovascular diseases	QFPDD and WMT	Multi	Non-RCT	Four gastrointestinal reactions occurred in three patients, no serious adverse events	Retrospective study	No deaths
Chen Y et al. (2024) [34]	CHD	Yangyin Jiedu Mixture and WMT	Single	RCT	No	Lack of longer follow-up	No cures or deaths were reported. After treatment, compared to control group, the total effective rate of the experimental group was higher (100% vs 87.5%), the experimental group showed a more significant decrease in ll-6, CRP, NlR, TCM symptom score and VAS score of chest pain, etc
Huang Z et al. (2020) [38]	HF	BADUANJIN combined with a modified Baoxin decoction and WMT	Single	RCT	No	Single-center	No cures or deaths were reported.After treatment, compared to control group, the total effective rate of the experimental group was higher, etc
Zong XY et al. (2022) [51]	Respiratory diseases	QFPDD and WMT	Multi	Non-RCT	No	A retrospective study with small sample size, no parallel control group	All patients were cured and discharged
Jiao LW et al. (2021) [43]	DM	QFPDD and WMT	Multi	Non-RCT	Gastrointestinal reactions occurred among three patients, no serious adverse events	Retrospective study	All patients were cured and discharged
Tao LW et al. (2023) [44]	DM	JYGBD and WMT	Single	RCT	No	Single-center	All patients wer cured and discharged. After treatment, the improvement of clinical symptoms in treatment group was significantly better than control group
Xu RL et al. (2023) [62]	DM and HBV	MXSGD, Sanren decoction, and WMT	Single	Case reports	No	Small sample size	Cured and discharged
Chen RB et al. (2021) [17]	HBV	QFPDD and WMT	Multi	Non-RCT	Three patients occurred gastrointestinal reactions in control group and two in treatment group, no serious adverse events	Small sample size	All patients were cured and discharged. (Duration of viral shedding in the treatment group was significantly shorter than control group, and the improvement of clinical symptoms was significantly better than control group.)
Yang WJ et al. (2023) [63]	Abdominal compartment syndrome	Dushen decoction, Xiao Chengqi decoction Juyuan decoction, acupuncture, and WMT	Single	Case reports	No	Small sample size	Cured and discharged
Huang W et al. (2022) [64]	CKD	Recommended formula 1-3 and WMT	Single	Non-RCT	No	Single-Center, retrospective study	6.5% patients in treatment group were died, 36.3% patients in control group were died. Compared with the control group, the mortality of the treatment group was significantly reduced, and after treatment the clinical symptoms and laboratory abnormal index were significantly improved.)
Cen J et al. (2023) [65]	CKD	JHQG, LHQW, Xuebijing, and WMT	Single	Non-RCT	One patient occurred nauseating, two occurred pruritus and 1 occurred diarrhea	Retrospective study	93.22% patients were cured and discharged. 6.78% patients died

*HF* heart failure, *CHD* coronary heart disease, *DM* Diabetes Mellitus, *HBV* hepatitis B, *CKD* chronic kidney disease, *RCT* randomized controlled trials, *QFPDD* Qingfei Paidu Decoction, *MXSGD* Maxing Shigan Decoction, *JYGBD* Jingyin Gubiao Decoction, *JHQG* Jinhua Qinggan granule, *LHQW* Lianhua Qingwen capsule (granule), *WMT* Western medicine treatments; ADR, adverse reaction

Recommended formula 1 includes Cang Zhu 15 g, Chen Pi 10 g, Hou Po 10 g, Huo Xiang 10 g, Cao Guo 6 g, Sheng Ma Huang 6 g, Qiang Huo 10 g, Sheng Jiang 10 g, and Bing Lang 10 g

Recommended formula 2 includes Sheng Ma Huang (EH) 6 g, Xing Ren 9 g, heng Shi Gao 15 g, Gan Cao 3 g, Huo Xiang10 g, Hou Po10 g, Cang Zhu 15 g, Cao Guo 10 g, Fa Ban Xia 9 g, Fu Ling 15 g, Sheng Da Huang 5 g, Sheng Huang Qi 10 g, Ting Li Zi 10 g, and Chi Shao10 g;

Recommended formula 3 includes Su He Xiang Wan or Angong Niuhuang Wan with the following decoction composed of Ren Shen 15 g, Hei Shun Pian 10 g, and Shan Zhu Yu 15 g

Five systemic diseases were included in our study, including COVID-19 with circulatory diseases such as hypertension, coronary heart disease and HF; COVID-19 with endocrine and metabolic diseases such as diabetes; COVID-19 with respiratory diseases such as pulmonary tuberculosis (PTB) and COPD; COVID-19 with digestive diseases such as hepatitis; COVID-19 with urogenital diseases and other comorbidities. An overview of COVID-19 with comorbidities was shown in Table 2.

**Table 2:** Overview of COVID-19 with comorbidities

Ref included certain comorbidities	System	Comorbidities
Zong et al. (2020)	Respiratory system	Pneumonia
Zong et al. (2020)		Tuberculosis
Zong et al. (2020)		Chronic obstructive pulmonary disease
Huang et al. (2020)	Circulatory system	Heart failure
Chen et al. (2024)		Coronary heart disease
Li et al. (2021)		Hypertension
Li et al. (2020)		
Zhang et al. (2021)		Diseases of blood vessel
Xu et al. (2023)	Digestive system	Hepatitis
Chen et al. (2021)		
Yang et al. (2023)		Abdominal compartment syndrome
Huang et al. (2022)	Urogenital system	Renal failure
Cen et al. (2023)		
Jiao et al. (2021)	Endocrine and metabolic system	Diabetes
Tao et al. (2023)		
Xu et al. (2023)		

Additionally, we conducted a quality assessment to 13 studies included. An evaluation of the literature showed that none of the three RCTs reported the use of allocation concealment, two mentioned randomized grouping, however the specific method of randomization was not described in detail thus scoring 3, and 1 explicitly described the method of randomization thus scoring 4. Two cohort studies scored 18, and five out of six observational studies scored 12 due to the absence of a control situation, with the additional one item was scored as 14 because it reported on follow-up. As for two case reports, except for the item “Were adverse reactions or unexpected events detected and described?” was evaluated as “no”, the rest of the evaluation items were “yes”. We considered that the non-RCTs studies enrolled were high and moderate studies, and RCTs were high and low studies (Tables 3, 4and5).

**Table 3:** Quality assessment of non-random control trials

Studies	MINORS evaluation criterion												Score	Quality
	(1)	(2)	(3)	(4)	(5)	(6)	(7)	(8)	(9)	(10)	(11)	(12)		
Huang et al. (2022)	2	2	2	2	0	0	2	0	2	2	2	2	18	High
Chen et al. (2021)	2	2	2	2	0	0	2	0	2	2	2	2	18	High
Zong et al. (2022)	2	2	2	2	0	0	2	0	0	0	0	2	12	Medium
Cen et al. (2023)	2	2	2	2	0	2	2	0	0	0	0	2	14	High
Li et al. (2020)	2	2	2	2	0	0	2	0	0	0	0	2	12	Medium
Li et al. (2021)	2	2	2	2	0	0	2	0	0	0	0	2	12	Medium
Jiao et al. (2021)	2	2	2	2	0	0	2	0	0	0	0	2	12	Medium
Zhang et al. (2021)	2	2	2	2	0	0	2	0	0	0	0	2	12	Medium

(1) A clearly stated aim; (2) Inclusion of consecutive patients; (3) Prospective collection of data; (4) Endpoints appropriate to the aim of the study; (5) Unbiased assessment of the study endpoint; (6) Follow-up period appropriate to the aim of the study; (7) Loss to follow up less than 5%; (8) Prospective calculation of the study size; (9) An adequate control group; (10) Contemporary groups; (11) Baseline equivalence of groups; (12) Adequate statistical analyses

**Table 4:** Quality assessment of random control trials

Studies	Jadad evaluation criterion				Score	Quality
	(1)	(2)	(3)	(4)		
Chen et al. (2024)	2	1	1	0	4	high
Huang et al. (2020)	1	1	1	0	3	low
Tao et al. (2023)	1	1	1	0	3	low

(1) Randomization; (2) Concealment of allocation; (3) Double blinding; (4) Withdrawals and dropouts

**Table 5:** Quality assessment of case reports

Studies	JBI evaluation criterion							
	(1)	(2)	(3)	(4)	(5)	(6)	(7)	(8)
Xu et al. (2023)	Yes	Yes	Yes	Yes	Yes	Yes	No	Yes
Yang et al. (2023)	Yes	Yes	Yes	Yes	Yes	Yes	No	Yes

(1) Were patient's demographic characteristics clearly described? (2) Was the patient's history clearly described and presented as a timeline? (3) Was the current clinical condition of the patient on presentation clearly described? (4) Were diagnostic tests or assessment methods and the results clearly described? (5) Was the intervention(s)or treatment procedure(s) clearly described? (6) Was the post-intervention clinical condition clearly described? (7) Were adverse events (harms) or unanticipated events identified and described? (8) Does the case report provide takeaway lessons?

### COVID-19 with diseases of circulatory system

The circulatory comorbidities of COVID-19 mainly include hypertension, coronary heart disease, and cerebrovascular disease. A total of 12.1% of the studies indicated that 18.7% of COVID-19 patients had circulatory comorbidities [25], with higher mortality and ICU occupancy rates [26]. A retrospective cohort study showed that COVID-19 patients with cardiovascular and cerebrovascular diseases had better outcomes and significantly improved clinical symptoms (cough, fatigue, asthma, fever, dry cough, etc.) after TCM treatment (QFPDD), and lung CT imaging indicated absorption of pulmonary lesions in 92.5% of patients without serious adverse reactions [20].

### Hypertension

Relevant studies indicated that hypertension is the most common comorbidity in COVID-19 patients, accounting for 45.8% [25], and is associated with adverse comprehensive outcomes, including death, ICU admission, and acute respiratory distress syndrome [27, 28]. In a study conducted among hospitalized COVID-19 patients in Iran, those with hypertension alone had a significantly higher mortality rate than those without comorbidities (20.8% vs. 12.7%) [29]. There are two clinical studies of TCM included in this disease.

A multicenter retrospective study showed that COVID-19 patients with hypertension received QFPDD in combination with Western medicine treatments (WMT) such as antiviral drugs, antibiotics. After six days of treatment, the clinical symptoms of different proportion such as fever and fatigue had been improved. 74.7% of the 99 cases with abnormal lung imaging results showed absorption of lung lesions; 99.2% (118/119) patients were cured and discharged. No serious adverse events occurred in all patients during hospitalization [16]. Li et al. found that the mean length of hospital stay for COVID-19 patients with hypertension was 10.8 ± 3.8 days, the mean systolic blood pressure decreased by 14.0 ± 5.7 mmHg, and the mean diastolic blood pressure decreased by 8.4 ± 2.5 mmHg using QFPDD with oxygen therapy and symptomatic supportive therapy without antiviral, anti-inflammatory, immune-modulating medications. These findings suggested that QFPDD could promote the negative conversion of SARS-CoV-2 RNA, facilitate the absorption of chest lesions, and help lower blood pressure in COVID-19 patients with hypertension [30]. Mihardja et al. demonstrated the efficacy and mechanism of acupuncture in the treatment of COVID-19 patients with hypertension and type 2 diabetes. The results indicated that acupuncture reduced blood pressure in patients with hypertension and lowered blood glucose and insulin resistance in patients with type 2 diabetes, suggesting that which might be a potential method to be used as an adjuvant therapy for such patients [31].

### Coronary heart disease and heart failure

The proportion of COVID-19 patients with coronary heart disease (CHD) ranges from 10.7% to 15.6% [25]. Previous study showed that compared to COVID-19 patients without comorbidities, those with a history of CHD had a 1.45-fold increased risk of death, 1.57-fold increased risk of developing severe or critical cases, 1.75-fold increased risk of ICU/CCU admission [32], and up to 10.5% of the overall mortality rate [33]. Previous study reported that the combination of Yangyin Jiedu Mixture with fluid infusion and conventional treatment significantly improved the degree of chest pain in patients (total effective rate,100.0% vs. 87.5%) and enhanced the efficacy of electrocardiograms (total effective rate, 90.3% vs. 68.8%), and lower TCM syndrome scores after treatment (6.0 ± 1.6 vs. 7.8 ± 2.2) compared to conventional treatment alone. Furthermore, laboratory indicators such as IL-6, C-reactive protein, and the neutrophil-to-lymphocyte ratio showed significant improvement [34].

The proportion of COVID-19 patients with HF ranges from 4.3 to 24% [25, 35], and HF increases the risk of myocardial injury and death in COVID-19 patients [36]. Previous study predicted that 20 active compounds and 164 targets of Shenfu injection, which were mainly involved in three biological processes of metabolism, coagulation and cytokine signaling pathways in the immune system, and play an important role in the prevention and treatment of COVID-19 complicated with HF. It is suggested that Shenfu injection might be the potential to treat COVID-19 combined with HF [37]. In addition, there is one clinical study of TCM included in this disease. A RCT showed that compared to control group, BADUANJIN is as a traditional Chinese exercise that emphasizes a harmonious connection between mind and body through slow, coordinated, and sequential movements combined with modified Baoxin decoction and Yiqi Fumai freeze-dried powder had higher effective rate in the experimental group (54.2% vs 33.3%). Left ventricular ejection fraction (42.4 ± 6.7 vs. 37.7 ± 7.1) and left ventricular end-systolic diameter (3.8 ± 1.1 vs. 4.4 ± 1.0) were significantly improved in the treatment group compared to the control group [38].

### Cerebrovascular diseases

The proportion of COVID-19 patients with cerebrovascular disease ranges from 1.0% to 1.9%, and cerebrovascular diseases increase the mortality rate of patients with COVID-19 [39]. A multicenter retrospective study reported that 96.3% of COVID-19 patients with stroke were treated by QFPDD, and 96.3% of patients were cured and discharged [40].

### COVID-19 with diseases of endocrine and metabolic system

COVID-19 co-infected endocrine and metabolic diseases include diabetes, obesity, malnutrition, thyroid dysfunction, and hyperlipidemia, among them, the most common was diabetes. These comorbidities increase the risk of death in COVID-19 patients.

### Diabetes

Studies indicated that 8.4–10.9% of COVID-19 patients had diabetes, which was the second most common comorbidity for COVID-19 [13]. The disease severity and mortality rate increased in COVID-19 patients with diabetes [41], with the mortality rate up to 7.8% [42]. A multicenter retrospective study showed that the number of fevers, cough and fatigue among COVID-19 patients with type 2 diabetes were significantly reduced at day 3 and day 6 during treatment compared to those before treatment, No serious adverse events occurred during treatment. All patients were cured and discharged from hospitals [43]. Tao et al. reported that Jingyin Gubiao decoction had significantly lowered TCM syndrome scores such as chills, sweating, expectoration, and cough *(P* < *0.05)* [44]. Besides, Hu et al. showed that *Anemarrhena asphodeloides *Bunge, *Astragalus membranaceus* (Fisch.) Bge., *Lonicera japonica* Thunb., and *Scutellaria baicalensis* Georgi were high-frequency medicines for COVID-19, and showed favorable effect of blood-glucose control, suggesting that these TCM have a promising perspective for the treatment of COVID-19 with diabetes based on 120 Chinese herbal formulas analyzed [45]. Furthermore, previous studies demonstrated that the effective control of blood glucose with hypoglycemic agents such as metformin, insulin, DPP-4 inhibitors were conducive to reduce the incidence of complications and the risk of death in patients. Studies also showed that the earlier use of hypoglycemic agents, the better treatment outcome [41, 42, 46].

### COVID-19 with diseases of respiratory system

Respiratory comorbidities associated with COVID-19 mainly include COPD and PTB. Studies indicated that about 5.2–9.0% of COVID-19 patients have other chronic lung diseases [25]. They also revealed that patients with respiratory diseases before infection were more prone to develop acute respiratory distress syndrome. These patients had poor prognosis due to their impaired lung function and reduced resistance to the virus [13, 47].

### Pulmonary tuberculosis

Studies indicated that 1.3–1.9% of COVID-19 patients have tuberculosis [48], and PTB may increase the mortality rate up to 13.0% in these patients [49, 50]. Clinical symptoms such as cough and fever of COVID-19 patients with PTB were significantly improved after receiving QFPDD and WMT such as antiviral drugs and antibiotics. One 67-year-old female patient had negative nucleic acid test result after 2 days of treatment with QFPDD and was discharged after 17 days of hospitalization. Another 72-year-old male patient negative nucleic acid test result after 4 days of QFPDD treatment and was discharged after 23 days of hospitalization [51].

### Chronic obstructive pulmonary disease

COPD is a common comorbidity of COVID-19, affecting approximately 6.0% of patients have COPD [52]. COPD is an independent risk factor for worsening symptoms and death in COVID-19 patients. It can increase the incidence of cardiovascular events, mechanical ventilation, hospitalization, and rehospitalization [53–56]. A case series study reported that QFPDD presented good efficacy to COVID-19 patient with COPD without adverse reactions. and clinical symptoms were improved significantly after 7 days of QFPDD treatment, and the patient was cured and discharged after 9 days of hospitalization [51].

### Other pulmonary infectious diseases

14.0–25.0% of hospitalized COVID-19 patients have other infections [57], and other pulmonary infectious diseases include community-acquired and hospital-acquired pneumonia. COVID-19 patients with other pulmonary infectious diseases have more severe symptoms and higher mortality rate. The most common causes are bacterial infections, fungal infections, and infections with influenza viruses, mycoplasma, and chlamydia. There is one clinical study of TCM included in this disease. A case series study showed that COVID-19 patients with lobar pneumonia and community-acquired pneumonia presented with significant improvement in symptoms such as fever, fatigue, cough, and asthma after treatment with QFPDD and conventional WMT such as antiviral drugs and antibiotics, and chest CT showed absorption of lung lesions without adverse reactions [51].

### COVID-19 with diseases of digestive system

COVID-19 with comorbidities across digestive system mainly include viral hepatitis including hepatitis A virus (HAV), hepatitis B virus (HBV), hepatitis C virus (HCV), hepatitis D virus (HDV), and hepatitis E virus (HEV), autoimmune hepatitis (AIH), fatty liver, liver cirrhosis, and pancreatitis. Among these, HBV is the most common digestive comorbidity. Digestive comorbidities can increase the mortality rate in COVID-19 patients [58]. Currently, there are few clinical studies on TCM. Studies have shown that COVID-19 patients with hepatitis have good outcomes after TCM treatment without adverse reactions.

### Hepatitis

Liver disease has attracted widespread attention, and approximately 3.0%–4.0% of COVID-19 patients have chronic hepatitis. Hepatitis B is the second (2.1%) comorbidity in COVID-19 patients with non-cardiometabolic diseases [13]. COVID-19 patients with chronic hepatitis not only face an increased risk of liver injury (incidence approximately 43.4%) and disease severity but also have higher mortality rate [13, 59–61]. Chen et al. reported that patients with fever, cough, dry cough, fatigue, sore throat and anorexia decreased more in QFPDD group than in conventional treatment group during 6 days of treatment. The time to negative conversion of nucleic acid test result of QFPDD group was 7.0 (5.5, 8.0) days, while that of conventional treatment group was 7.5(5.5, 10.0) days, with statistically significant difference between groups (*P* = 0.013) [17]. Another study reported a 44-year-old female COVID-19 patient with HBV who has received TCM and WMT such as antiviral drugs and antibiotics, after 13-days treatment, symptoms such as chest tightness, dry mouth, frequent hunger were improved, and lung lesions were significantly aborted, finally was cured and discharged [62]. Currently, no relevant reports on the use of TCM for treating COVID-19 with HCV, HDV, HEV, or AIH are available.

### Abdominal compartment syndrome

A case report of TCM for abdominal compartment syndrome was included in this paper. Yang et al. presented a patient with COVID-19 combined abdominal compartment syndrome. After received TCM including Dushen decoction, Xiao Chengqi decoction, and Juyuan decoction, etc. Inflammation of lungs were significantly absorbed and gastrointestinal motility was recovery [63].

### COVID-19 with diseases of urogenital system

The urogenital system comorbidities of COVID-19 mainly include chronic kidney disease (CKD). Studies showed that 4.8%−6.6% of COVID-19 patients have CKD, which increases the risk of renal injury and death in COVID-19 patients [25]. There are two clinical studies of TCM included in this disease. Huang et al. reported that TCM combined with WMT effectively could improve symptoms such as anorexia, reduce D-dimer levels, increase the recovery rate, and significantly decrease the mortality rate among COVID-19 patients with CKD. In the treatment group, 6.5% of patients died, compared to 36.3% of patients in the control group. No serious adverse reactions were reported [64]. Cen et al. observed that COVID-19 patients with CKD were treated with TCM and WMT such as hemodialysis, clinical symptoms including cough, sputum, fever, diarrhea, and poor appetite were improved. Abnormal laboratory indicators of inflammation including serum leukocyte counts, erythrocyte sedimentation rate, C-reactive protein, and lymphocytes were all improved. Finally, 94.92% patients were discharged from the hospital the average length of hospital stay was 16.8 ± 6.1 days [65].

## Discussion

In the present study, we conducted a comprehensive and systematic review on TCM in fighting COVID-19 with comorbidities across various systems such as circulatory system, endocrine and metabolic, respiratory system, digestive system, and urogenital system. 13 clinical studies were enrolled totally including 3 RCTs, 8 non-RCTs, and 2 case reports. Existing studies indicated TCM had good efficacy and safety in treatment of COVID-19 with comorbidities, the findings could provide reference for clinical application.

### TCM showed favourable treatment outcome for COVID-19 with comorbidities

Clinical studies on the treatment of COVID-19 with comorbidities by TCM were focused on diseases of circulatory system [16, 20, 30, 37], including hypertension, HF, and CHD; diseases of endocrine and metabolic system, including diabetes [17]; diseases of respiratory system [51], including PTB and COPD; diseases of digestive system, including hepatitis [17]; and diseases of urogenital system. Based on the current studies on medication, QFPDD is the most widely used TCM, and related clinical studies showed that QFPDD could effectively improve the clinical symptoms of patients of COVID-19 with hypertension, cardiovascular and cerebrovascular diseases, PTB, COPD, diabetes and HBV. Additionally, studies demonstrated that Yangyin Jiedu Mixture, BADUANJIN combined with a modified Baoxin decoction, and Yiqi Fumai freeze-dried powder combined with WMT could effectively treat COVID-19 with CHD and HF [34, 38]. Jingyin Gubiao decoction also could improve the clinical symptoms of COVID-19 patients with diabetes [44]. JHQG, LHQW, Xuebijing combined with WMT had better effects in treating COVID-19 patients with urinary diseases than using WMT alone [64, 65]. Notably, no serious adverse reactions were reported for the TCM mentioned. The findings indicated that TCM for COVID-19 patients with comorbidities of different disease systems had favorable treatment outcome.

Despite clinical studies demonstrated that TCM presented favorable treatment outcome. Whereas, TCM had different effectiveness in treating COVID-19 with comorbidities of different disease systems. The possible reasons are related following factors. Firstly, different study design such as different study population and area, single or multi-center; prospective or retrospective study [16, 20]; Secondly, Chinese herbal formulas are composed of multiple herbs based on the principles of “sovereign, minister, assistant, and courier” under the guidance of TCM theory. Each formula is tailored to target specific organs and pathogenesis, and the organs and pathogenesis involved in different diseases often different. This variation can be resulted in different therapeutic effects of the same TCM formula when treating comorbidities across different systems [66].

### Current research gaps in TCM for COVID-19 with comorbidities

Other systemic comorbidities of COVID-19 include rheumatic, hematological, reproductive, nervous, skin diseases, and malignant tumors. Currently, no clinical studies of TCM were reported. Previous study indicated that the proportion of COVID-19 patients with malignant tumors ranges from 1.9% to 3.5% [67]. Cancer is classified as a solid and hematological malignant tumor, and COVID-19 patients with malignant tumors significantly increased mortality and ICU occupancy rates [68]. Studies showed that COVID-19 patients with rheumatic diseases, anemia, and nervous system diseases have an increased incidence of adverse outcomes [9, 11, 69, 70]. In COVID-19 patients, rheumatic diseases, rheumatoid arthritis, gout, and systemic lupus erythematosus are the most common comorbidities, accounting for 48.2%, 14.4%, and 8.1%, respectively, with an overall mortality rate of 9.0% [71]. Thus, specific attention should be paid to data concerning COVID-19 patients with above multiple underlying diseases such as rheumatic, hematological systemic diseases. Multi-center studies should be conducted in the future, and provide high-level evidence to support the use of TCM in managing COVID-19 with various comorbidities. Further address gaps in the modern research field.

## Strengths and limitations

There are some notable strengths in the current study. First, to the best of our knowledge, this is the first study to explore efficacy of TCM in treatment of COVID-19 with comorbidities across various systems. Second, we found that COVID-19 patients co-infected comorbidities had favorable treatment outcome with TCM, suggesting that TCM has great application prospects in treating COVID-19 with comorbidities. Despite the strengths of the present study, there are still some limitations. First, the literature search was restricted to Chinese and English databases, and databases from Japan and Korea were not searched, which may introduce language bias. Additionally, nearly 80% studies were observational studies, the results might be bias, the reason is most studies were conducted in the early of COVID-19 pandemic, due to the urgency of treating patients and the situation of prevention and control, this might bring certain challenges to conduct multi-center RCTs.

## Conclusion

In summary, TCM in fighting COVID-19 with comorbidities mostly focused on circulatory diseases, followed by endocrine and metabolic diseases, respiratory diseases. TCM has significantly improved the clinical symptoms such as fever, fatigue, dry cough, anorexia and asthma, the absorption of lung lesions, shortened the duration of viral shedding and the course of disease among COVID-19 patients with comorbidities, and without serious adverse reactions. The findings presented that TCM presented good efficacy and safety, indicates TCM could be effective way. Additionally, we also found that no related studies on TCM and COVID-19 with rheumatic, hematological, reproductive and nervous diseases, etc. More high-quality studies should be conducted in the future, and could help addressing gaps, thereby providing references to prevent and treat COVID-19 with comorbidities across various systems for clinicians.

## Supplementary Information

Supplementary Material 1

## Data Availability

Not applicable.

## Abbreviations

COVID-19: Corona Virus Disease 2019
SARS-CoV-2: Severe acute respiratory syndrome coronavirus 2
TCM: Traditional Chinese medicine
WMT: Western medicine treatments
WHO: World Health Organization
RCTs: Randomized controlled trials
ACEIs: Angiotensin-converting enzyme inhibitors
ARBs: Angiotensin receptor blockers
CHD: Coronary heart disease
HF: Heart failure
DM: Diabetes mellitus
HBV: Hepatitis B
CKD: Chronic kidney disease
QFPDD: Qingfei Paidu decoction
JYGBD: Jingyin Gubiao decoction
JHQG: Jinhua Qinggan granule
LHQW: Lianhua Qingwen capsule (granule)
ADR: Adverse reaction
COPD: Chronic obstructive pulmonary disease
PTB: Pulmonary tuberculosis
