# Supplementary material for: Recent advancements in traditional Chinese medicine for COVID-19 with comorbidities across various systems: a scoping review
Source: Infect Dis Poverty. 2024 Dec 19;13:97. doi: 10.1186/s40249-024-01263-8 (PMC11658301; doi:10.1186/s40249-024-01263-8)
Supplement: Supplementary file 1 — Supplementary Material 1 [file 40249_2024_1263_MOESM1_ESM.docx]

**Additional files. Additional tables.**

**Additional file 1. Search strategies in each database.**

**Table S1-A: Search terms used in English bibliographic databases**

| **Database** | **Search cluster** | **Search terms** |
| --- | --- | --- |
| Web of Science | Traditional Chinese Medicine | (Huashi baidu OR HSBD[Title/Abstract] OR Xuanfei baidu[Title/Abstract] OR XFBD[Title/Abstract] OR Jinhua Qinggan[Title/Abstract] OR JHQG[Title/Abstract] OR Lianhua Qingwen [Title/Abstract] OR LHQW[Title/Abstract] OR Xuebijing[Title/Abstract] OR XBJ[Title/Abstract] OR Qingfei Paidu Decoction[Title/Abstract] OR Lung Cleansing & detoxifying Decoction[Title/Abstract] OR Lung Cleansing detoxifying decoction[Title/Abstract] OR QFPDD[Title/Abstract] OR traditional Chinese medicine[Title/Abstract] OR TCM[Title/Abstract] OR oriental medicine[Title/Abstract] OR Chinese Medicine[Title/Abstract]) AND |
|  | COVID-19 | (COVID-19[Title/Abstract] OR covid 19[Title/Abstract] OR sars cov 2[Title/Abstract] OR coronavirus 2019[Title/Abstract] OR coronavirus disease 2019[Title/Abstract] OR Novel coronavirus[Title/Abstract] OR 2019-ncov[Title/Abstract]) AND |
|  | Treatment | (treatment[Title/Abstract] OR effective[Title/Abstract] OR effect[Title/Abstract] OR outcome[Title/Abstract] OR follow up[Title/Abstract] OR efficacy[Title/Abstract] OR therapeutic effect[Title/Abstract]) AND |
| PubMed | Traditional Chinese Medicine | (Huashi baidu OR HSBD[Title/Abstract] OR Xuanfei baidu[Title/Abstract] OR XFBD[Title/Abstract] OR Jinhua Qinggan[Title/Abstract] OR JHQG[Title/Abstract] OR Lianhua Qingwen [Title/Abstract] OR LHQW[Title/Abstract] OR Xuebijing[Title/Abstract] OR XBJ[Title/Abstract] OR Qingfei Paidu Decoction[Title/Abstract] OR Lung Cleansing & detoxifying Decoction[Title/Abstract] OR Lung Cleansing detoxifying decoction[Title/Abstract] OR QFPDD[Title/Abstract] OR traditional Chinese medicine[Title/Abstract] OR TCM[Title/Abstract] OR oriental medicine[Title/Abstract] OR Chinese Medicine[Title/Abstract]) AND |
|  | COVID-19 | (COVID-19[Title/Abstract] OR covid 19[Title/Abstract] OR sars cov 2[Title/Abstract] OR coronavirus 2019[Title/Abstract] OR coronavirus disease 2019[Title/Abstract] OR Novel coronavirus[Title/Abstract] OR 2019-ncov[Title/Abstract]) AND |
|  | Treatment | (treatment[Title/Abstract] OR effective[Title/Abstract] OR effect[Title/Abstract] OR outcome[Title/Abstract] OR follow up[Title/Abstract] OR efficacy[Title/Abstract] OR therapeutic effect[Title/Abstract]) AND |

**Table S1-B: Search terms used in Chinese bibliographic databases**

| **Database** | **Search cluster** | **Search terms** |
| --- | --- | --- |
| China National Knowledge Infrastructure | Traditional Chinese Medicine | 题名或关键词="金花清感颗粒"OR 题名或关键词="连花清瘟胶囊"OR 题名或关键词"连花清瘟颗粒”OR 题名或关键词"血必净注射液"OR 题名或关键词="清肺排毒汤"OR 题名或关键词="清肺排毒颗粒”OR 题名或关键词="化湿败毒方"OR 题名或关键词="化湿败毒颗粒"OR 题名或关键词="宣肺败毒方”OR 题名或关键词="宣肺败毒颗粒"OR 题名或关键词="中医药"OR 题名或关键词="中药")AND |
|  | COVID-19 | (题名或关键词="2019 冠状病毒"OR 题名或关键词="新型冠状病毒"OR 题名或关键词="新冠肺炎”OR 题名或关键词="新型冠状病毒肺炎"OR 题名或关键词="新型冠状病毒感染")AND |
|  | Treatment | (题名或关键词="疗效"OR 题名或关键词="治疗"OR 题名或关键词="转归”OR 题名或关键词="效果") AND |
| WANFANG | Traditional Chinese Medicine | 题名或关键词="金花清感颗粒"OR 题名或关键词="连花清瘟胶囊"OR 题名或关键词"连花清瘟颗粒”OR 题名或关键词"血必净注射液"OR 题名或关键词="清肺排毒汤"OR 题名或关键词="清肺排毒颗粒”OR 题名或关键词="化湿败毒方"OR 题名或关键词="化湿败毒颗粒"OR 题名或关键词="宣肺败毒方”OR 题名或关键词="宣肺败毒颗粒"OR 题名或关键词="中医药"OR 题名或关键词="中药")AND |
|  | COVID-19 | (题名或关键词="2019 冠状病毒"OR 题名或关键词="新型冠状病毒"OR 题名或关键词="新冠肺炎”OR 题名或关键词="新型冠状病毒肺炎"OR 题名或关键词="新型冠状病毒感染")AND |
|  | Treatment | (题名或关键词="疗效"OR 题名或关键词="治疗"OR 题名或关键词="转归”OR 题名或关键词="效果") AND |

**Additional file 2: Table S2** Composition of TCM

| **Studies** | **Comorbidities** | **Prescription** | **Chinese name** | **English name** | **Pinyin name** | **Latin name** | **Authorities** | **Family** | **Dose** | **Tissue used** |  |
| --- | --- | --- | --- | --- | --- | --- | --- | --- | --- | --- | --- |
|  |  |  |  |  |  |  |  |  |  |  |  |
| Jiao LW et al (2021) | DM | QFPDD | 麻黄 | Ephedrae Herba | Ma Huang | *Ephedra sinica* Stapf | *Ephedra sinica Stapf* | *Ephedraceae* | 9g | Herbaceous stem |  |
|  |  |  | 细辛 | Asari Radix et Rhizoma | Xi Xin | *Asarum heterotropoides* Fr. Schmidt var. *mandshuricum* (Maxim.)Kitag. | *Asarum heterotropoides F.Schmidt* | *Aristolochiaceae* | 6g | Roots and rhizomes |  |
|  |  |  | 桂枝 | Cinnamomi Ramulus | Gui Zhi | *Cinnamomum cassia* Presl | *Cinnamomum cassia (L.) J.Presl* | *Lauraceae* | 9g | Young shoot |  |
|  |  |  | 广藿香 | Pogostemonis Herba | Guang Huo Xiang | *Pogostemon cablin* (Blanco) Benth. | *Pogostemon cablin (Blanco) Benth.* | *Lamiaceae* | 9g | Aerial part |  |
|  |  |  | 白术 | Atractylodis Macrocephalae Rhizoma | Bai Zhu | *Atractylodes macrocephala* Koidz. | *Atractylodes macrocephala Koidz.* | *Asteraceae* | 9g | Rhizomes |  |
|  |  |  | 山药 | Dioscoreae Rhizoma | Shan Yao | *Dioscorea opposita* Thunb. | *Dioscorea oppositifolia L.* | *Dioscoreaceae* | 12g | Rhizomes |  |
|  |  |  | 石膏 | Gypsum Fibrosum | Shi Gao | *Gypsum Fibrosum* | *Gypsum Fibrosum* | *Sulfates* | 15g | - |  |
|  |  |  | 柴胡 | Bupleuri Radix | Chai Hu | *Bupleurum chinense* DC. | *Bupleurum chinense DC.* | *Apiaceae* | 16g | Roots |  |
|  |  |  | 苦杏仁 | Armeniacae Semen Amarum | Ku Xing Ren | *Prunus armeniaca* L. | *Prunus armeniaca L.* | *Rosaceae* | 9g | Seed |  |
|  |  |  | 款冬花 | Farfarae flos | Kuan Dong Hua | *Tussilago farfara* L. | *Tussilago farfara L.* | *Asteraceae* | 9g | Flower |  |
|  |  |  | 紫菀 | Asteris Radix et Rhizoma | Zi Wan | *Aster tataricus* L. f. | *Aster tataricus L.f.* | *Asteraceae* | 9g | Roots and rhizomes |  |
|  |  |  | 射干 | Belamcandae Rhizoma | She Gan | *Belamcanda chinensis*（L.）DC. | *Iris domestica (L.) Goldblatt & Mabb.* | *Iridaceae* | 9g | Rhizomes |  |
|  |  |  | 半夏 | Pinellia ternat | Ban Xia | *Pinellia ternata* (Thunb.) Breit. | *Pinellia ternata (Thunb.) Makino* | *Araceae* | 9g | Tuber |  |
|  |  |  | 生姜 | Zingiber officinale Roscoe | Sheng Jiang | *Zingiber oficinale* Rosc. | *Zingiber officinale Roscoe* | *Zingiberaceae* | 9g | Rhizomes |  |
|  |  |  | 枳实 | Aurantii Immaturus Fructus | Zhi Shi | *Citrus aurantium* L. | *Citrus × aurantium f. aurantium* | *Rutaceae* | 6g | Immature fruit |  |
|  |  |  | 陈皮 | Citri Reticulatae Pericarpium | Chen Pi | *Citrus reticulata* Blanco | *Citrus reticulata Blanco* | *Rutaceae* | 6g | Fruit peel |  |
|  |  |  | 猪苓 | Polyporus | Zhu Ling | *Polyporus umbellatus*（Pers.）Fries | *Polyporus* | *Sargassaceae* | 9g | Sclerotium |  |
|  |  |  | 茯苓 | Poria | Fu Ling | *Poria cocos* (Schw. ) Wolf | *Poria* | *Polyporaceae* | 15g | Sclerotium |  |
|  |  |  | 泽泻 | Alismatis Rhizoma | Ze Xie | *Alisma orientate* （Sam.） Juzep. | *Alisma plantago-aquatica subsp. orientale (Sam.) Sam.* | *Alismataceae* | 9g | Tuber |  |
|  |  |  | 黄芩 | Scutellariae Radix | Huang Qin | *Scutellaria baicalensis* Georgi | *Scutellaria baicalensis Georgi* | *Lamiaceae* | 6g | Roots |  |
|  |  |  | 炙甘草 | Glycyrrhizae Radix et Rhizoma Praeparata Cum Melle | Zhi Gan Cao | *Glycyrrhiza uralensis* Fisch. | *Glycyrrhiza uralensis Fisch. ex DC.* | *Fabaceae* | 6g | Roots and rhizomes |  |
| Zhang L et al (2021) | Cardiovascular  and  CerebrovaScular   Diseases | QFPDD | Same to study(Jiao LW et al (2021)) | | | | | | | |  |
| Zong XY et al (2020) | Respiratory   Diseases | QFPDD | Same to study(Jiao LW et al (2021)) | | | | | | | |  |
| Li HZ et al (2021) | Hypertension | QFPDD | Same to study(Jiao LW et al (2021)) | | | | | | | |  |
| Chen RB etal (2021) | HBV | QFPDD | Same to study(Jiao LW et al (2021)) | | | | | | | |  |
| Chen Y et al (2024) | CHD | Yangyin Jiedu Mixture | 北沙参 | Coastal Glehnia Root | Bei Shashen | *Glehnia littoralis* F. Schmidt ex Miq. | *Glehnia littoralis (A.Gray) F.Schmidt ex Miq.* | Apiaceae | NR | Roots |  |
|  |  |  | 石斛 | Noble Dendrobium Stem Herb | Shi Hu | *Dendrobium nobile* Lindl. | *Dendrobium nobile Lindl.* | *Orchidaceae* | NR | Stem |  |
|  |  |  | 玉竹 | Fragrant Solomonseal Rhizome | Yu Zhu | *Polygonatum odoratum* (Mill.) Druce | *Polygonatum odoratum (Mill.) Druce* | *Asparagaceae* | NR | Rhizomes |  |
|  |  |  | 石菖蒲 | Acorus Tatarinowii | Shi Chang Pu | *Acorus calamus* L. | *Acorus calamus var. angustatus Besser* | *Acoraceae* | NR | Rhizomes |  |
|  |  |  | 绵马贯众 | Dryopteris Crassirhizoma | Mianma Guanzhong | *Dryopteris crassirhizoma* Nakai | *Dryopteris crassirhizoma Nakai* | *Polypodiaceae* | NR | Rhizomes |  |
|  |  |  | 苍术 | Rhizoma Atractylodis | Cang Zhu | *Atractylodes lancea* (Thunb.) DC. | *Atractylodes lancea (Thunb.) DC.* | *Asteraceae* | NR | Rhizomes |  |
|  |  |  | 泽兰 | lycopus lucidus | Ze Lan | *Eupatorium japonicum* Thunb. | *Chromolaena odorata (L.) R.M.King & H.Rob.* | *Asteraceae* | NR | Aerial part |  |
| Li YZ et al (2020) | Hypertension | Qingfei Decoction | 瓜蒌皮 | Trichosanthis Fructus | Gua Lou Pi | *Trichosanthes kirilowii* Maxim. | *Trichosanthes kirilowii Maxim.* | *Cucurbitaceae* | 10g | Fruit peel |  |
|  |  |  | 清半夏 | Pinellia ternat | Qing Ban Xia | *Pinellia ternata* (Thunb.) Ten. ex Breitenb. | *Pinellia ternata (Thunb.) Makino* | *Araceae* | 15g | Tuber |  |
|  |  |  | 黄芩 | Scutellariae Radix | Huang Qin | *Scutellaria baicalensis* Georgi | *Scutellaria baicalensis Georgi* | *Lamiaceae* | 10g | Roots |  |
|  |  |  | 炙百部 | Stemonae Radix | Zhi Bai Bu | *Stemona japonica* (Blume) Miq. | *Stemona japonica (Blume) Miq.* | *Stemonaceae* | 10g | Roots |  |
|  |  |  | 前胡 | Peucedani Radix | Qian Hu | *Peucedanum praeruptorum* Dunn | *Kitagawia praeruptora (Dunn) Pimenov* | *Apiaceae* | 10g | Roots |  |
|  |  |  | 白前 | Cynanchi Stauntonii Rhizoma et Radix | Bai Qian | *Vincetoxicum glaucescens* (Decne.) C. Y. Wu & D. Z. Li | *Vincetoxicum stauntonii (Decne.) C.Y.Wu & D.Z.Li* | *Apocynaceae* | 10g | Roots and rhizomes |  |
|  |  |  | 炙甘草 | Glycyrrhizae Radix et Rhizoma | Zhi Gan Cao | *Glycyrrhiza uralensis* Fisch. | *Glycyrrhiza uralensis Fisch. ex DC.* | *Fabaceae* | 10g | Roots and rhizomes |  |
| Huang Z et al (2020) | HF | Chinese medicine formula | 人参 | Ginseng Radix et Rhizoma | Ren Shen | *Panax ginseng* C. A. Mey. | *Panax ginseng C. A. Mey.* | *Araliace* | 20g | Roots and rhizomes |  |
|  |  |  | 黄芪 | Astragali Radix | Huang Qi | *Astragalus membranaceus* (Fisch.) Bunge | *Astragalus mongholicus Bunge* | *Fabaceae* | 15g | Roots |  |
|  |  |  | 桂枝 | Cinnamomi Ramulus | Gui Zhi | *Cinnamomum cassia* Presl | *Cinnamomum cassia (L.) J.Presl* | *Lauraceae* | 10g | Young shoot |  |
|  |  |  | 茯苓 | Poria | Fu Ling | *Poria cocos*(Schw.)Wolf | *Poria* | *Polyporaceae* | 15g | Sclerotium |  |
|  |  |  | 猪苓 | Polyporus | Zhu Ling | *Polyporus umbellatus*(Pers.) Fries | *Polyporus* | *Sargassaceae* | 15g | Sclerotium |  |
|  |  |  | 泽泻 | Alismatis Rhizoma | Ze Xie | *Alisma orientate* （Sam.） Juzep. | *Alisma plantago-aquatica subsp. orientale (Sam.) Sam.* | *Alismataceae* | 15g | Tuber |  |
|  |  |  | 红花 | Carthami Flos | Hong Hua | *Carthamus tinctorius* L. | *Carthamus tinctorius L.* | *Asteraceae* | 20g | Flower |  |
|  |  |  | 川芎 | Chuanxiong Rhizoma | Chuan Xiong | *Ligusticum sinense 'Chuanxiong'* | *Conioselinum anthriscoides 'Chuanxiong'* | *Apiaceae* | 20g | Rhizomes |  |
| Tao LW et al (2023) | DM | JYGBD | 金银花 | Lonicerae Japonicae Flos | Jin Yin Hua | *Lonicera japonica* Thunb. | *Lonicera japonica Thunb.* | *Caprifoliaceae* | 9g | Flower |  |
|  |  |  | 荆芥 | Schizonepetae Herba | Jing Jie | *Nepeta cataria* L. | *Nepeta cataria L.* | *Lamiaceae* | 9g | Aerial part |  |
|  |  |  | 黄芪 | Astragali Radix | Huang Qi | *Astragalus membranaceus* (Fisch.) Bunge | *Astragalus mongholicus Bunge* | *Fabaceae* | 12g | Roots |  |
|  |  |  | 广藿香 | Pogostemonis Herba | Guang Huo Xiang | *Pogostemon cablin* (Blanco) Benth. | *Pogostemon cablin (Blanco) Benth.* | *Lamiaceae* | 9g | Aerial part |  |
|  |  |  | 防风 | Saposhnikoviae Radix | Fang Feng | *Saposhnikovia divaricata* (Turcz.) Schischk. | *Saposhnikovia divaricata (Trucz.) Schischk.* | *Apiaceae* | 9g | Roots |  |
|  |  |  | 板蓝根 | Isatidis Radix | Ban Lan Gen | *Isatis tinctoria* L. | *Isatis tinctoria subsp. tinctoria* | *Brassicaceae* | 9g | Roots |  |
|  |  |  | 桔梗 | Platycodon grandiflorus (Jacq.) A.DC. | Jie Geng | *Platycodon grandiflorus* (Jacq.) A. DC. | *Platycodon grandiflorus (Jacq.) A.DC.* | *Campanulaceae* | 6g | Roots |  |
|  |  |  | 芦根 | Phragmitis Rhizoma | Lu Gen | *Phragmites australis* (Cav.) Trin. ex Steud. | *Phragmites australis (Cav.) Trin. ex Steud.* | *Poaceae* | 15g | Rhizomes |  |
|  |  |  | 炒白术 | Atractylodis Macrocephalae Rhizoma | Chao Bai Zhu | *Atractylodes macrocephala* Koidz. | *Atractylodes macrocephala Koidz.* | *Asteraceae* | 9g | Rhizomes |  |
|  |  |  | 甘草 | Glycyrrhizae Radix et Rhizoma | Gan Cao | *Glycyrrhiza uralensis* Fisch. | *Glycyrrhiza uralensis Fisch. ex DC.* | *Fabaceae* | 9g | Roots and rhizomes |  |
| Yang WJ et al (2023) | Abdominal Compartment Syndrome | Chinese medicine formula 1 | 人参 | Ginseng Radix et Rhizoma | Ren Shen | *Panax ginseng* C. A. Mey. | *Panax ginseng C. A. Mey.* | *Araliaceae* | 30g | Roots and rhizomes |  |
|  |  |  | 玄明粉 | Natrii Sulfas Exsiccatus | Xuan Ming Fen | *-* | *Mangifera indica L.* | *Anacardiaceae* | 50g | - |  |
|  |  | Chinese medicine formula 2 | 人参 | Ginseng Radix et Rhizoma | Ren Shen | *Panax ginseng* C. A. Mey. | *Panax ginseng C. A. Mey.* | *Araliaceae* | 30g | Roots and rhizomes |  |
|  |  |  | 玄明粉 | Natrii Sulfas Exsiccatus | Xuan Ming Fen | *-* | *Mangifera indica L.* | *Anacardiaceae* | 30g | - |  |
|  |  | Chinese medicine formula 3 | 枳实 | Aurantii Immaturus Fructus | Zhi Shi | *Citrus aurantium* L. | *Citrus × aurantium f. aurantium* | *Rutaceae* | 30g | Immature fruit |  |
|  |  |  | 厚朴 | Magnoliae Officinalis Cortex | Hou Po | *Houpoea officinalis* (Rehder & E. H. Wilson) N. H. Xia & C. Y. Wu | *Magnolia officinalis Rehder & E.H.Wilson* | *Magnoliaceae* | 30g |  |  |
|  |  |  | 制大黄 | Rhei Radix et Rhizoma | Zhi Dai Huang | *Rheum palmatum* L. | *Rheum palmatum L.* | *Polygonaceae* | 15g | Roots and rhizomes |  |
|  |  |  | 人参 | Ginseng Radix et Rhizoma | Ren Shen | *Panax ginseng* C. A. Mey. | *Panax ginseng C. A. Mey.* | *Araliaceae* | 60g | Roots and rhizomes |  |
|  |  |  | 黄芪 | Astragali Radix | Huang Qi | *Astragalus membranaceus* (Fisch.) Bunge | *Astragalus mongholicus Bunge* | *Fabaceae* | 60g | Roots |  |
|  |  |  | 炒白术 | Atractylodis Macrocephalae Rhizoma | Chao Bai Zhu | *Atractylodes macrocephala* Koidz. | *Atractylodes macrocephala Koidz.* | *Asteraceae* | 30g | Rhizomes |  |
| Huang W et al (2022) | CKD | Chinese medicine formula 4 | 苍术 | Rhizoma Atractylodis | Cang Zhu | *Atractylodes lancea* (Thunb.) DC. | *Atractylodes lancea（Thunb.）DC.* | *Asteraceae* | 15g | Rhizomes |  |
|  |  |  | 陈皮 | Citri Reticulatae Pericarpium | Chen Pi | *Citrus reticulata* Blanco | *Citrus reticulata Blanco* | *Rutaceae* | 10g | Fruit peel |  |
|  |  |  | 厚朴 | Magnoliae Officinalis Cortex | Hou Po | *Houpoea officinalis* (Rehder & E. H. Wilson) N. H. Xia & C. Y. Wu | *Magnolia officinalis Rehder & E.H.Wilson* | *Magnoliaceae* | 10g |  |  |
|  |  |  | 藿香 | Agastache rugosa | Huo Xiang | *Pogostemon cablin* (Blanco) Benth. | *Pogostemon cablin (Blanco) Benth.* | *Lamiaceae* | 10g | Aerial part |  |
|  |  |  | 草果 | Amomum tsaoko Crevost et Lemarie | Cao Guo | *Amomum tsaoko* Crevost & Lem. | *Lanxangia tsao-ko (Crevost & Lemarié) M.F.Newman & Škorničk.* | *Zingiberaceae* | 6g | Immature fruit |  |
|  |  |  | 生麻黄 | Ephedrae Herba | Sheng Ma Huang | *Ephedra sinica* Stapf | *Ephedra sinica Stapf* | *Ephedraceae* | 6g | Herbaceous stem |  |
|  |  |  | 羌活 | Notopterygii Rhizoma et Radix | Qiang Huo | *Hansenia weberbaueriana* (Fedde ex H. Wolff) Pimenov & Kljuykov | *Hansenia weberbaueriana (Fedde ex H.Wolff) Pimenov & Kljuykov* | *Apiaceae* | 10g | Roots and rhizomes |  |
|  |  |  | 生姜 | Zingiber officinale Roscoe | Sheng Jiang | *Zingiber oficinale* Rosc. | *Zingiber officinale Roscoe* | *Zingiberaceae* | 10g | Tuber |  |
|  |  |  | 槟榔 | Arecae Semen | Bing Lang | *Areca catechu* L. | *Areca catechu L.* | *Arecaceae* | 10g | Seed |  |
|  | CKD | Chinese medicine formula 5 | 杏仁 | Armeniacae Semen Amarum | Xing Ren | *Tussilago farfara* L. | *Prunus armeniaca L.* | *Rosaceae* | 10g | Seed |  |
|  |  |  | 生石膏 | Gypsum Fibrosum | Sheng Shi Gao | *Gypsum Fibrosum* | *Gypsum Fibrosum* | *Sulfates* | 30g | - |  |
|  |  |  | 瓜蒌 | Trichosanthis Fructus | Gua Lou | *Trichosanthes kirilowii* Maxim. | *Trichosanthes kirilowii Maxim.* | *Cucurbitaceae* | 30g | Immature fruit |  |
|  |  |  | 生大黄 | Rhei Radix et Rhizoma | Sheng Dai Huang | *Rheum palmatum* L. | *Rheum palmatum L.* | *Polygonaceae* | 6g | Roots and rhizomes |  |
|  |  |  | 生麻黄 | Ephedrae Herba | Sheng Ma Huang | *Ephedra sinica* Stapf | *Ephedra sinica Stapf* | *Ephedraceae* | 6g | Herbaceous stem |  |
|  |  |  | 炙麻黄 | Ephedrae Herba | Zhi Ma Huang | *Ephedra sinica* Stapf | *Ephedra sinica Stapf* | *Ephedraceae* | 6g | Herbaceous stem |  |
|  |  |  | 葶苈子 | Descurainiae Semen | Ting Li Zi | *Stellaria alsine* Grimm | *Descurainia sophia（L.）Webb. ex Prantl.* | *Brassicaceae* | 10g | Seed |  |
|  |  |  | 桃仁 | Persicae Semen | Tao Ren | *Prunus persica* (L.) Batsch | *Prunus persica (L.) Batsch* | *Rosaceae* | 10g | Seed |  |
|  |  |  | 草果 | Amomum tsaoko Crevost et Lemarie | Cao Guo | *Amomum tsaoko* Crevost & Lem. | *Lanxangia tsao-ko (Crevost & Lemarié) M.F.Newman & Škorničk.* | *Zingiberaceae* | 6g | Immature fruit |  |
|  |  |  | 槟榔 | Arecae Semen | Bing Lang | *Areca catechu* L. | *Areca catechu L.* | *Arecaceae* | 10g | Seed |  |
|  |  |  | 苍术 | Rhizoma Atractylodis | Cang Zhu | *Atractylodes lancea* (Thunb.) DC. | *Atractylodes lancea（Thunb.）DC.* | *Asteraceae* | 10g | Rhizomes |  |
|  | CKD | Chinese medicine formula 6 | 人参 | Ginseng Radix et Rhizoma | Ren Shen | *Panax ginseng* C. A. Mey. | *Panax ginseng C. A. Mey.* | *Araliaceae* | 15g | Roots and rhizomes |  |
|  |  |  | 黑顺片 | Aconiti Lateralis Radix Praeparata | Hei Shun Pian | *Aconitum carmichaelii* Debeaux | *Aconitum carmichaelii Debeaux* | *Ranunculaceae* | 10g | Roots |  |
|  |  |  | 山茱萸 | Corni Fructus | Shan Zhu Yu | *Cornus officinalis* Siebold & Zucc. | *Cornus officinalis Siebold & Zucc.* | *Cornaceae* | 15g | Immature fruit |  |
| Cen J et al (2023) | CKD | Chinese medicine formula 7 | 苍术 | Rhizoma Atractylodis | Cang Zhu | *Atractylodes lancea* (Thunb.) DC. | *Atractylodes lancea（Thunb.）DC.* | *Asteraceae* | 15g | Rhizomes |  |
|  |  |  | 陈皮 | Citri Reticulatae Pericarpium | Chen Pi | *Citrus reticulata* Blanco | *Citrus reticulata Blanco* | *Rutaceae* | 10g | Fruit peel |  |
|  |  |  | 厚朴 | Magnoliae Officinalis Cortex | Hou Po | *Houpoea officinalis* (Rehder & E. H. Wilson) N. H. Xia & C. Y. Wu | *Magnolia officinalis Rehder & E.H.Wilson* | *Magnoliaceae* | 10g | Trunk, roots and shoot bark |  |
|  |  |  | 藿香 | Agastache rugosa | Huo Xiang | *Pogostemon cablin* (Blanco) Benth. | *Pogostemon cablin (Blanco) Benth.* | *Lamiaceae* | 10g | Roots and rhizomes |  |
|  |  |  | 草果 | Amomum tsaoko Crevost et Lemarie | Cao Guo | *Amomum tsaoko* Crevost & Lem. | *Lanxangia tsao-ko (Crevost & Lemarié) M.F.Newman & Škorničk.* | *Zingiberaceae* | 6g | Immature fruit |  |
|  |  |  | 生麻黄 | Ephedrae Herba | Sheng Ma Huang | *Ephedra sinica* Stapf | *Ephedra sinica Stapf* | *Ephedraceae* | 6g | Herbaceous stem |  |
|  |  |  | 羌活 | Notopterygii Rhizoma et Radix | Qiang Huo | *Hansenia weberbaueriana* (Fedde ex H. Wolff) Pimenov & Kljuykov | *Hansenia weberbaueriana (Fedde ex H.Wolff) Pimenov & Kljuykov* | *Apiaceae* | 10g | Roots and rhizomes |  |
|  |  |  | 生姜 | Zingiber officinale Roscoe | Sheng Jiang | *Zingiber oficinale* Rosc. | *Zingiber officinale Roscoe* | *Zingiberaceae* | 10g | Tuber |  |
|  |  |  | 槟榔 | Arecae Semen | Bing Lang | *Areca catechu* L. | *Areca catechu L.* | *Arecaceae* | 10g | Seed |  |
|  | CKD | Chinese medicine formula | 杏仁 | Armeniacae Semen Amarum | Xing Ren | *Tussilago farfara* L. | *Prunus armeniaca L.* | *Rosaceae* | 10g | Seed |  |
|  |  |  | 生石膏 | Gypsum Fibrosum | Sheng Shi Gao | *Gypsum Fibrosum* | *Gypsum Fibrosum* | *Sulfates* | 30g | - |  |
|  |  |  | 瓜蒌 | Trichosanthis Fructus | Gua Lou | *Trichosanthes kirilowii* Maxim. | *Trichosanthes kirilowii Maxim.* | *Cucurbitaceae* | 30g | Immature fruit |  |
|  |  |  | 生大黄 | Rhei Radix et Rhizoma | Sheng Dai Huang | *Rheum palmatum* L. | *Rheum palmatum L.* | *Polygonaceae* | 6g | Roots and rhizomes |  |
|  |  |  | 生麻黄 | Ephedrae Herba | Sheng Ma Huang | *Ephedra sinica* Stapf | *Ephedra sinica Stapf* | *Ephedraceae* | 6g | Herbaceous stem |  |
|  |  |  | 炙麻黄 | Ephedrae Herba | Zhi Ma Huang | *Ephedra sinica* Stapf | *Ephedra sinica Stapf* | *Ephedraceae* | 6g | Herbaceous stem |  |
|  |  |  | 葶苈子 | Descurainiae Semen | Ting Li Zi | *Stellaria alsine* Grimm | *Descurainia sophia（L.）Webb. ex Prantl.* | *Brassicaceae* | 10g | Seed |  |
|  |  |  | 桃仁 | Persicae Semen | Tao Ren | *Prunus persica* (L.) Batsch | *Prunus persica (L.) Batsch* | *Rosaceae* | 10g | Seed |  |
|  |  |  | 草果 | Amomum tsaoko Crevost et Lemarie | Cao Guo | *Amomum tsaoko* Crevost & Lem. | *Lanxangia tsao-ko (Crevost & Lemarié) M.F.Newman & Škorničk.* | *Zingiberaceae* | 6g | Immature fruit |  |
|  |  |  | 槟榔 | Arecae Semen | Bing Lang | *Areca catechu* L. | *Areca catechu L.* | *Arecaceae* | 10g | Seed |  |
|  |  |  | 苍术 | Rhizoma Atractylodis | Cang Zhu | *Atractylodes lancea* (Thunb.) DC. | *Atractylodes lancea（Thunb.）DC.* | *Asteraceae* | 10g | Rhizomes |  |
|  |  |  | 姜黄 | Curcumae Longae Rhizoma | Jiang Huang | *Curcuma Longa* L. | *Curcuma longa L.* | *Zingiberaceae* | 9g | Rhizomes |  |
|  |  |  | 僵蚕 | Bombyx Batryticatus | Jiang Can | *Bombyx mori* Linnaeus | *Bombyx mori Linnaeus* | *Silkworm pilgrimaging* | 9g | - |  |
|  | CKD | Chinese medicine formula | 法半夏 | Pinelliae Rhizoma | Fa Ban Xia | *Pinellia ternata* (Thunb.) Ten. ex Breitenb. | *Pinellia ternata (Thunb.) Makino* | *Araceae* | 9g | Tuber |  |
|  |  |  | 陈皮 | Citri Reticulatae Pericarpium | Chen Pi | *Citrus reticulata* Blanco | *Citrus reticulata Blanco* | *Rutaceae* | 10g | Fruit peel |  |
|  |  |  | 党参 | Codonopsis Radix | Dang Shen | *Codonopsis pilosula* (Franch.) Nannf. | *Codonopsis pilosula(Franch.) Nannf.* | *Campanulaceae* | 15g | Roots |  |
|  |  |  | 炙黄芪 | Astragali Radix | Zhi Huang Qi | *Astragalus membranaceus* (Fisch.) Bunge | *Astragalus mongholicus Bunge* | *Fabaceae* | 30g | Roots |  |
|  |  |  | 茯苓 | Poria | Fu Ling | *Poria cocos* (Schw. ) Wolf | *Poria* | *Polyporaceae* | 15g | Sclerotium |  |
|  |  |  | 藿香 | Agastache rugosa | Huo Xiang | *Pogostemon cablin* (Blanco) Benth. | *Pogostemon cablin (Blanco) Benth.* | *Lamiaceae* | 10g | Aerial part |  |
|  |  |  | 砂仁 | Amomi Fructus | Sha Ren | *Amomum villosum* Lour. | *Amomum villosum Lour.* | *Zingiberaceae* | 6g | Immature fruit |  |
|  | CKD | Chinese medicine formula | 南沙参 | Adenophorae Radix | Nan Sha Shen | *Adenophora tetraphylla* (Thunb.) Fisch. | *Adenophora triphylla (Thunb.) A.DC.* | *Campanulaceae* | 15g | Roots |  |
|  |  |  | 人参 | Ginseng Radix et Rhizoma | Ren Shen | *Panax ginseng* C. A. Mey. | *Panax ginseng C. A. Mey.* | *Araliaceae* | 9g | Roots and rhizomes |  |
|  |  |  | 天冬 | Asparagi Radix | Tian Dong | *Asparagus cochinchinensis* (Lour.) Merr. | *Asparagus cochinchinensis (Lour.) Merr.* | *Asparagaceae* | 9g | Root tuber |  |
|  |  |  | 麦冬 | Ophiopogon japonicus(Linn. f.)Ker-Gawl. | Mai Dong | *Ophiopogon japonicus* (L. f.) Ker Gawl. | *Ophiopogon japonicus (Thunb.) Ker Gawl.* | *Asparagaceae* | 9g | Root tuber |  |
|  |  |  | 淡竹叶 | Lophatheri Herba | Dan Zhu Ye | *Lophatherum gracile* Brongn*.* | *Lophatherum gracile Brongn.* | *Poaceae* | 9g | Stem and leaves |  |
|  |  |  | 桑叶 | Mori Folium | Sang Ye | *Morus alba* L. | *Morus alba L.* | *Moraceae* | 9g | Leaves |  |
|  |  |  | 蝉衣 | Cicadae Periostracum | Chan Tui | *Cryptotympana pustulata* Fabricius | *Cryptotympana pustulata Fabricius* | *Cicadidae* | 6g | - |  |
|  |  |  | 地骨皮 | Lycii Cortex | Di Gu Pi | *Lycium chinense* Mill. | *Lycium shawii Roem. & Schult.* | *Solanaceae* | 9g | Roots peel |  |
|  |  |  | 炒苍术 | Rhizoma Atractylodis | Chao Cang Zhu | *Atractylodes lancea* (Thunb.) DC. | *Atractylodes lancea（Thunb.）DC.* | *Asteraceae* | 15g | Rhizomes |  |
|  |  |  | 炒谷芽 | Setariae Germinatus Fructus | Cha Gu Ya | *Oryza sativa* L*.* | *Oryza sativa L.* | *Poaceae* | 15g | Immature fruit |  |
| Xu RL et al (2023) | DM and HBV | Chinese medicine formula | 麻黄 | Ephedrae Herba | Ma Huang | *Ephedra sinica* Stapf | *Ephedra sinica Stapf* | *Ephedraceae* | 6g | Herbaceous stem |  |
|  |  |  | 杏仁 | Armeniacae Semen Amarum | Xing Ren | *Tussilago farfara* L. | *Prunus armeniaca L.* | *Rosaceae* | 10g | Seed |  |
|  |  |  | 石膏 | Gypsum Fibrosum | Shi Gao | *Gypsum Fibrosum* | *Gypsum Fibrosum* | *Sulfates* | 15g | - |  |
|  |  |  | 薏苡仁 | Coicis Semen | Yi Yi Ren | *Coix lacryma-*jobi var. ma-yuen (Rom. Caill.) Stapf | *Coix lacryma-jobi var. ma-yuen (Rom.Caill.) Stapf* | *Poaceae* | 30g | Seed |  |
|  |  |  | 砂仁 | Amomi Fructus | Sha Ren | *Amomum villosum* Lour. | *Amomum villosum Lour.* | *Zingiberaceae* | 10g | Immature fruit |  |
|  |  |  | 厚朴 | Magnoliae Officinalis Cortex | Hou Po | *Houpoea officinalis* (Rehder & E. H. Wilson) N. H. Xia & C. Y. Wu | *Magnolia officinalis Rehder & E.H.Wilson* | *Magnoliaceae* | 10g | Trunk, roots and shoot bark |  |
|  |  |  | 半夏 | Pinellia ternata | Ban Xia | *Pinellia ternata* (Thunb.) Ten. ex Breitenb. | *Pinellia ternata (Thunb.) Makino* | *Araceae* | 10g | Tuber |  |
|  |  |  | 生姜 | Zingiber officinale Roscoe | Sheng Jiang | *Zingiber oficinale* Rosc. | *Zingiber officinale Roscoe* | *Zingiberaceae* | 10g | Rhizomes |  |
|  |  |  | 石菖蒲 | Acori Tatarinowii Rhizoma | Shi Chang Pu | *Acorus calamus* L. | *Acorus gramineus Aiton* | *Acoraceae* | 10g | Rhizomes |  |
|  |  |  | 连翘 | Forsythia suspensa | Lian Qiao | *Forsythia suspensa* (Thunb.) Vahl | *Forsythia suspensa （Thunb.） Vahl* | *Oleaceae* | 10g | Immature fruit |  |
|  |  |  | 白术 | Atractylodis Macrocephalae Rhizoma | Bai Zhu | *Atractylodes macrocephala* Koidz. | *Atractylodes macrocephala Koidz.* | *Asteraceae* | 10g | Rhizomes |  |
|  |  |  | 藿香 | Agastache rugosa | Guang Huo Xiang | *Pogostemon cablin* (Blanco) Benth. | *Pogostemon cablin (Blanco) Benth.* | *Lamiaceae* | 10g | Aerial part |  |
|  |  |  | 葶苈子 | Descurainiae Semen | Ting Li Zi | *Stellaria alsine* Grimm | *Descurainia sophia（L.）Webb. ex Prantl.* | *Brassicaceae* | 15g | Seed |  |
|  |  |  | 陈皮 | Citri Reticulatae Pericarpium | Chen Pi | *Citrus reticulata* Blanco | *Citrus reticulata Blanco* | *Rutaceae* | 10g | Fruit peel |  |
|  |  |  | 甘草 | Glycyrrhiza uralensis Fisch. | Gan Cao | *Glycyrrhiza uralensis* Fisch*.* | *Glycyrrhiza uralensis Fisch. ex DC.* | *Fabaceae* | 10g | Roots and rhizomes |  |
|  | DM and HBV | Chinese medicine formula | 薏苡仁 | Coicis Semen | Yi Yi Ren | *Coix lacryma-*jobi var. ma-yuen (Rom. Caill.) Stapf | *Coix lacryma-jobi var. ma-yuen (Rom.Caill.) Stapf* | *Poaceae* | 30g | Seed |  |
|  |  |  | 白豆蔻 | Amomi Fructus Rotundus | Bai Dou Kou | *Amomum kravanh* Pierre ex Gagnep. | *Wurfbainia compacta (Sol. ex Maton) Škorničk. & A.D.Poulsen* | *Zingiberaceae* | 10g | Immature fruit |  |
|  |  |  | 杏仁 | Armeniacae Semen Amarum | Ku Xing Ren | *Prunus armeniaca* L. | *Prunus armeniaca L.* | *Rosaceae* | 10g | Seed |  |
|  |  |  | 草果 | Amomum tsaoko Crevost et Lemarie | Cao Guo | *Amomum tsaoko* Crevost & Lem. | *Lanxangia tsao-ko (Crevost & Lemarié) M.F.Newman & Škorničk.* | *Zingiberaceae* | 10g | Immature fruit |  |
|  |  |  | 厚朴 | Magnoliae Officinalis Cortex | Hou Po | *Houpoea officinalis* (Rehder & E. H. Wilson) N. H. Xia & C. Y. Wu | *Magnolia officinalis Rehder & E.H.Wilson* | *Magnoliaceae* | 10g | Trunk, roots and shoot bark |  |
|  |  |  | 生姜 | Zingiber officinale Roscoe | Sheng Jiang | *Zingiber oficinale* Rosc. | *Zingiber officinale Roscoe* | *Zingiberaceae* | 10g | Rhizomes |  |
|  |  |  | 槟榔 | Arecae Semen | Bing Lang | *Areca catechu* L. | *Areca catechu L.* | *Arecaceae* | 10g | Seed |  |
|  |  |  | 石菖蒲 | Acorus Tatarinowii | Shi Chang Pu | *Acorus calamus* L. | *Acorus calamus var. angustatus Besser* | *Acoraceae* | 10g | Rhizomes |  |
|  |  |  | 滑石 | Talc | Hua Shi | *Magnesium* silicate | *Talcum* | *Silicate* | 15g | - |  |
|  |  |  | 甘草 | Glycyrrhiza uralensis Fisch. | Gan Cao | *Glycyrrhiza uralensis* Fisch*.* | *Glycyrrhiza uralensis Fisch. ex DC.* | *Fabaceae* | 6g | Roots and rhizomes |  |
|  |  |  | 白术 | Atractylodis Macrocephalae Rhizoma | Bai Zhu | *Atractylodes macrocephala* Koidz. | *Atractylodes macrocephala Koidz.* | *Asteraceae* | 15g | Rhizomes |  |
|  |  |  | 半夏 | Pinellia ternata | Ban Xia | *Pinellia ternata* (Thunb.) Ten. ex Breitenb. | *Pinellia ternata (Thunb.) Makino* | *Araceae* | 10g | Tuber |  |
|  |  |  | 葶苈子 | Descurainiae Semen | Ting Li Zi | *Stellaria alsine* Grimm | *Descurainia sophia（L.）Webb. ex Prantl.* | *Brassicaceae* | 30g | Seed |  |
|  |  |  | 赤芍 | Red paeony root | Chi Shao | *Paeonia lactiflora* Pall | *Paeonia lactiflora Pall* | *Roots* | 20g | Roots |  |
|  |  |  | 牡丹皮 | Tree Peony Bark | Mu Dan Pi | *Paeonia × suffruticosa* Andrews | *Paeonia × suffruticosa* Andrews | *Root bark* | 20g | Root barks |  |
|  |  |  | 黄芩 | Scutellariae Radix | Huang Qin | *Scutellaria baicalensis* Georgi | *Scutellaria baicalensis Georgi* | *Lamiaceae* | 15g | Roots |  |
|  |  |  | 黄连 | Coptis chinensis | Huang Lian | *Coptis chinensis* Franch. | *Coptis chinensis Franch* | *Ranunculaceae* | 6g | Rhizomes |  |
|  |  |  | 大黄 | Rhei Radix et Rhizoma | Zhi Dai Huang | *Rheum palmatum* L. | *Rheum palmatum L.* | *Polygonaceae* | 5g | Roots and rhizomes |  |

Note: HF, heart failure; CHD, coronary heart disease; DM, Diabetes Mellitus; HBV, hepatitis B; CKD, chronic kidney disease; QFPDD, Qingfei Paidu Decoction; JYGBD, Jingyin Gubiao Decoction.

NR indicates not record data. Study of Chen Y et al (2024) reported Yangyin Jiedu Mixture, was released as a TCM preparation for COVID-19 on official website of the Guizhou Provincial Administration of Traditional Chinese Medicine(<http://atcm.guizhou.gov.cn/xwzx/ttxw/202003/t20200304_53165921.html>), whereas, the specific dosage was not announced, and the reference did not provide the dosage of each herb in the study.

Usages of each TCM:

(1) **Jiao LW et al. (2021)**. QFPDD, traditional Chinese medicine decoction pieces for water decoction. Administer one dose per day, twice in the morning and evening (forty minutes after a meal), with warm water, three doses per course of treatment, and complete two treatment courses in the study;

(2) **Zhang L et al. (2021). QEPDD,** same to study (Jiao LW et al (2021);

(3) **Zong XY et al. (2020)****. QEPDD,** same to study (Jiao LW et al (2021);

(4) **Li HZ et al. (2021). QEPDD,** same to study (Jiao LW et al (2021)

(5) **Chen RB et al. (2021). QEPDD,** same to study (Jiao LW et al (2021)

(6) **Chen Y et al. (2024).** Yangyin Jiedu Mixture, oral, 3 times a day, 15 ml each time, the course of treatment is 7 days

(7) **Li YZ et al. (2020).** Qingfei Decoction, Granules, melted with water, 1 dose a day, twice in the morning and evening, and the course of treatment is two weeks

(8) **Huang Z et al. (2020).** Chinese medicine formula, traditional Chinese medicine decoction pieces for water decoction. Administer one dose per day, twice in the morning and evening, one treatment course is 7 days, and continuous treatment of 3 courses.

(9) **Tao LW et al. (2023). JYGBD,** granules, 1 package a day, twice in the morning and evening, and the course of treatment is two weeks;

(10) **Yang WJ et al. (2023)**. Chinese medicine formula1, traditional Chinese medicine decoction pieces for water decoction.

Administer one dose per day, decocting water for 100mL juice, divided into 5 times of slow nasal feeding, 1time per hours, and treatment courses is 3 doses. **Chinese medicine formula** 2- 3 is same to Chinese medicine formula1.

(11) **Huang W et al. (2022)**. **Chinese medicine formula**, traditional Chinese medicine decoction pieces for water decoction. Administer one dose per day, twice in the morning and evening, one treatment course is 7 days, and continuous treatment of 3 courses.

(12) **Cen J et al. (2023). Chinese medicine formula,** traditional Chinese medicine decoction pieces for water decoction. Administer one dose per day, twice in the morning and evening.

(13) **Xu J et al. (2023)**. **Chinese medicine formula**, traditional Chinese medicine decoction pieces for water decoction. Administer one dose per day, twice in the morning and evening.
